# Factors Associated With Rebleeding and Early Mortality Following Transcatheter Arterial Embolization for Spontaneous Muscle Hematoma: A Single-Center Experience Including the Period of the Coronavirus Disease Pandemic

**DOI:** 10.7759/cureus.76347

**Published:** 2024-12-24

**Authors:** Toshihiro Horii, Mitsuhiro Kishino, Koji Morishita, Eiichiro Kanda, Marie Takahashi, Koichiro Kimura, Takuya Adachi, Jun Oyama, Sayuri Okawa, Ukihide Tateishi

**Affiliations:** 1 Diagnostic Radiology and Nuclear Medicine, Tokyo Medical and Dental University, Tokyo, JPN; 2 Diagnostic Radiology, National Cancer Center Hospital East, Chiba, JPN; 3 Trauma and Acute Care Surgery, Tokyo Medical and Dental University Hospital, Tokyo, JPN; 4 Medical Science, Kawasaki Medical School, Okayama, JPN

**Keywords:** covid-19, early mortality, rebleeding, spontaneous muscle hematoma, transcatheter arterial embolization

## Abstract

Objectives

We aim to investigate factors associated with rebleeding and mortality within one month of transcatheter arterial embolization (TAE) for spontaneous muscle hematoma (SMH) and the impact of the novel coronavirus disease 2019 (COVID-19).

Methods

This retrospective analysis included 33 patients who underwent TAE for SMH at a single center between 2012 and 2022. After 2020, eight of these patients had the COVID-19 infection. Patient characteristics, laboratory findings, embolic materials, and imaging findings were compared between the rebleeding and non-rebleeding groups, as well as between the early mortality and survival groups.

Results

Among all patients, 72.7% were on anticoagulant therapy before the onset of SMH. Of these, 27.2% required retreatment due to rebleeding. Patients who experienced rebleeding were more likely to have a platelet count below 50,000/µL, fibrinogen levels below 150 mg/dL, and an activated partial thromboplastin time (APTT) ratio above 2.5. Patients with SMH unrelated to anticoagulants had a higher rebleeding rate (56%), which may serve as a predictor of rebleeding. No significant difference in rebleeding rates was observed between patients with and without COVID-19 infection.

Early mortality within one month of onset occurred in 24.2% of patients, with a higher prevalence among those with a history of malignancy. However, there was no increase in early mortality among patients who required retreatment for rebleeding.

Conclusions

Patients with a low platelet count, fibrinogen level, prolonged APTT, and non-anticoagulant-related SMH are at a high risk of rebleeding and require close monitoring. Severe comorbidities, including malignancies and COVID-19, can affect mortality rates. TAE remained effective even in cases of rebleeding.

Advances in knowledge

This study indicated non-anticoagulant-related SMH and hematological parameters as factors associated with rebleeding.

## Introduction

Spontaneous muscle hematoma (SMH) is an intramuscular bleed that occurs spontaneously without a trigger such as trauma or medical injury. Although rare, SMH can sometimes be fatal [1]. Its primary anatomic sites include the abdominal wall, iliopsoas, and gluteal regions [2]. Anticoagulation therapy has been identified as a risk factor for the development of SMH [3,4], and its incidence in patients receiving anticoagulation therapy has been reported to be 0.6% [2]. With the increasing number of patients receiving anticoagulation therapy, SMH has become more prevalent [5,6].

Additionally, the emergence of severe acute respiratory syndrome coronavirus 2, a novel coronavirus disease 2019 (COVID-19) infection, since 2019 has added a new dimension to the management of SMH [7]. Anticoagulation therapy is recommended for patients with severe COVID-19 infection owing to the increased risk of venous thromboembolism (VTE) [8-10]. However, in addition to the increased VTE risk, the risk of bleeding complications is also increased in patients with COVID-19. Abate et al. reported a 2.1% incidence of SMH among 475 COVID-19-infected hospitalized patients [11].

Although research on the treatment of SMH is limited, transcatheter arterial embolization (TAE) has shown promise, particularly in severe cases [12]. However, high rates of rebleeding and mortality after embolization have also been reported [5,6,13,14]. There is limited understanding of the factors contributing to these outcomes, particularly in the case of rebleeding. We hypothesize that these refractory outcomes are associated with multiple patient-related factors rather than technical ones, and this study aims to identify the factors associated with rebleeding and mortality within one month of TAE for SMH. Additionally, we evaluated the impact of COVID-19 on SMH treatment.

## Materials and methods

Study design and population

**Figure 1:**
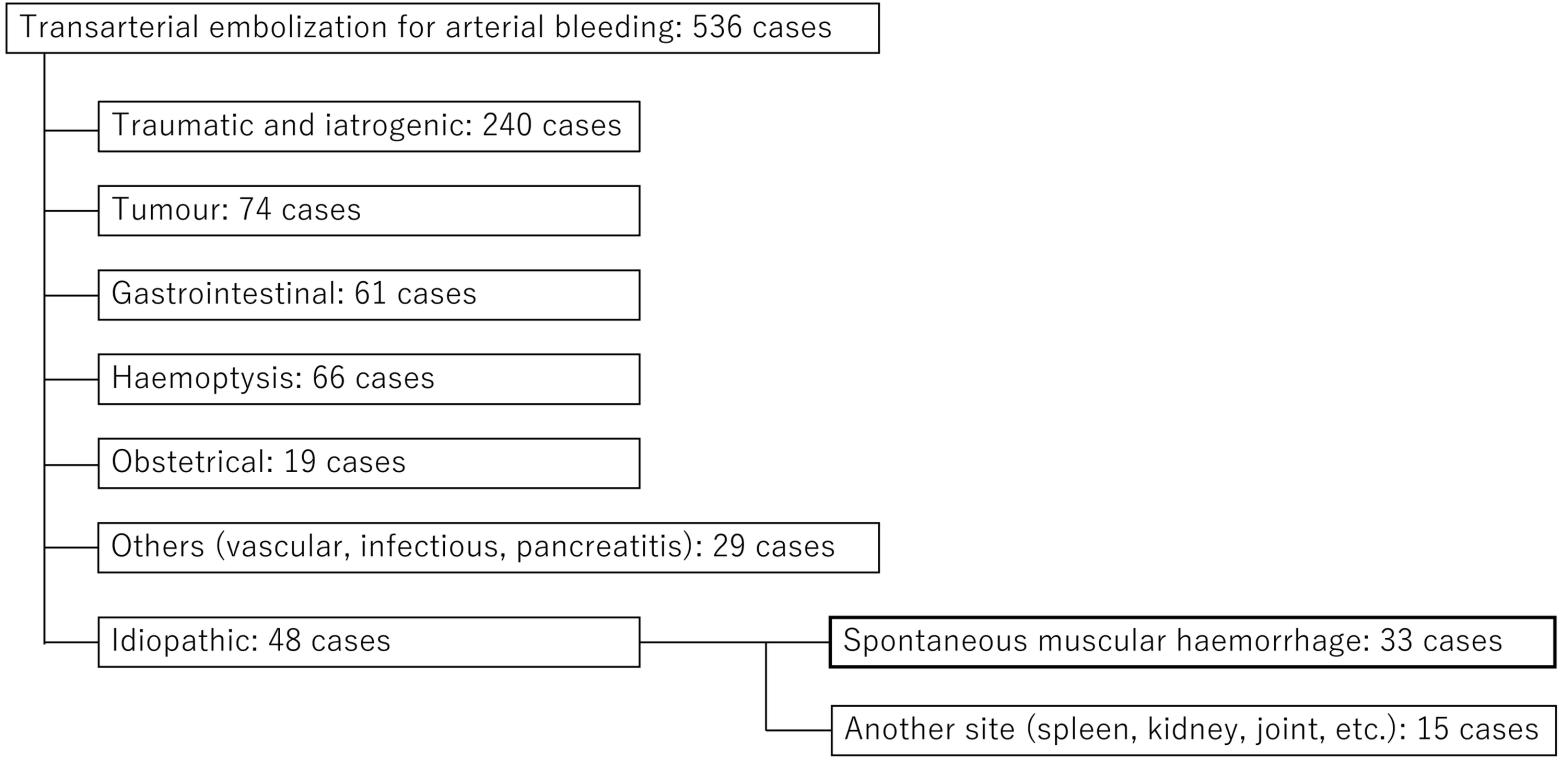
Breakdown of the etiology of patients who underwent transcatheter arterial embolization for arterial bleeding between 2012 and 2022

This is a retrospective observational study conducted in a single center. Of 536 patients who underwent TAE for hemostasis at our institution between January 2012 and December 2022, 33 patients diagnosed with SMH were included, and patients with other hemorrhagic lesions were excluded (Figure 1).

An SMH was defined as an intramuscular hematoma that developed without any apparent trigger and was unrelated to trauma, medical injury, local infection, or vascular lesions. Since 2020, COVID-19-related SMH cases have surged owing to an increased number of patient admissions due to severe COVID-19 [11].

The indication for TAE was determined through a multidisciplinary discussion involving an interventional radiologist and a physician. This decision was based on active bleeding observed on contrast-enhanced computed tomography (CECT) and/or persistent hemodynamic instability. Of the 33 patients, 27 underwent multiphase CT, two underwent single-phase CECT, and two with severe COVID-19 were assessed using interventional radiology CT (IR-CT) in the angiography suite. In one case, CECT was omitted due to renal dysfunction, and in another, plain CT via IR-CT was performed for rapid assessment in a patient with severe COVID-19 and hypotension. TAE was performed in all cases after confirming contrast extravasation via angiography or IR-CT.

TAE protocol

Embolization was performed using an IR-CT-equipped angiography system (Toshiba Infinix Celeve-I INFX-8000C; Aquilion TSX-201A, Otawara, Japan). The femoral artery approach was performed with patients under local anesthesia. A 4- to 5-French (Fr) introducer sheath was also used. Initially, dynamic subtraction angiography was used to identify vessels with active bleeding. If necessary, IR-CT was used to localize the exact bleeding site for embolization. After a thorough search for potential bleeding vessels, selective embolization was performed using a 1.9- to 2.9-Fr microcatheter, and prophylactic embolization was added at the operator's discretion.

The choice of the embolization material was based on the operator's clinical decision. Patients were categorized into two groups according to the embolic materials used: those treated solely with gelatine microspheres and those treated with either coils or N-butyl cyanoacrylate (NBCA). If coils or NBCA were used with gelatine, the patients were assigned to the coil or NBCA group. Technical success was confirmed by successful embolization with no contrast leakage observed on angiography.

Data collection

We collected pre-treatment patient information from electronic medical and treatment records. In addition to COVID-19 infection, we collected patient background characteristics potentially involved in bleeding susceptibility with reference to the Academic Research Consortium for High Bleeding Risk (ARC-HBR) criteria [15], which are international assessment criteria for high bleeding risk in patients undergoing percutaneous coronary intervention defined by the Academic Research Consortium in 2019; these assessment criteria are based on accumulating recent evidence and practicality. Data on the following risk factors for bleeding were collected: age, sex, previous cerebral or cardiovascular treatment within three months, previous surgery within three months, active malignancy within 12 months, dialysis, cirrhosis with portal hypertension, previous steroid therapy, oral antiplatelet agent use, oral anticoagulant use, and heparin therapy. The following blood test values were collected from the final pre-treatment measurements: hemoglobin level, platelet count, activated partial thromboplastin time (APTT) ratio, prothrombin time/international normalized ratio (PT-INR), fibrinogen level, and estimated glomerular filtration rate (eGFR). Hematoma volume was measured using SYNAPSE VINCENT (Fujifilm Medical Co., Ltd., Tokyo, Japan), a medical image analysis system that provides three-dimensional visualization and volume assessment. The hematoma volume was calculated by subtracting the volume of the hematoma from the contralateral muscle volume without a hematoma. If the hematoma was located at multiple locations, the individual volumes were summed.

Post-treatment outcomes were also collected from the medical records. Patients who underwent re-embolization for rebleeding within one month of the initial treatment were classified into the rebleeding group. Re-embolization was performed if CECT revealed contrast medium extravasation. Patients who died within one month after the initial TAE, regardless of the reason, were included in the early mortality group.

Overall survival (OS) was calculated from the date of the first TAE to the date of the last follow-up or patient death during the study period.

Comparative analysis of rebleeding and early mortality

We compared patient background, history and medications, blood test values, and post-treatment course between the rebleeding and non-rebleeding groups. The blood test values were compared using predefined threshold values, as referenced from the European Trauma Guidelines, to assess the potential bleeding risk [16]. The threshold values were set as follows: hemoglobin value < 6 mg/dL, platelet count < 5×10^4^/μL, fibrinogen value < 150 mg/dL, PT-INR > 1.5, and APTT ratio > 2.5. Additionally, oral anticoagulants and heparin were combined and considered as a single factor in the anticoagulation group.

Subsequently, the proportion of rebleeding and the same aforementioned parameters were compared between the early mortality and survival groups.

Statistical analysis

The proportions of categorical data were evaluated using the Fisher exact test, and continuous variables were compared using the Mann-Whitney U test. The Kaplan-Meier method was used to estimate OS, and survival differences were analyzed using the log-rank test with Bonferroni correction. Statistical analyses were performed using R software for Windows version 4.2.2 (R Foundation, Vienna, Austria). Statistical significance was set at P<0.05.

Manuscript preparation

Some parts of this manuscript were revised using AI tools.

## Results

Patient characteristics and COVID-19-associated SMH

Technical success was achieved in all 33 patients who underwent TAE with no major complications. Table 1 shows the patients' characteristics, medical history, and laboratory values. Of the 33 patients, six were on oral anticoagulants and 18 were on heparin; moreover, 24 (72.7%) developed SMH while on anticoagulant therapy. The main reasons for taking oral anticoagulants were heart valve replacement in three patients, atrial fibrillation in two, and vasculitis in one. The main reasons for heparin use were COVID-19 in eight patients, VTE in six, and arterial embolism and/or atrial fibrillation in four. Cirrhosis with portal hypertension did not occur in any patient.

**Table 1:** Patient characteristics *The fibrinogen value was not available in one case. APTTR: activated partial thromboplastin time ratio, PT-INR: prothrombin time/international normalized ratio, eGFR: estimated glomerular filtration rate

Variables	Number (%)/median (range)
Age (years), median (range)	72 (43, 86)
Male sex, number (%)	22 (66)
Bleeding muscle, number (%)	
Iliopsoas	14 (42)
Gluteus and thigh wall	8 (24)
Thoracic and abdominal wall	7 (21)
Two regions	4 (12)
Volume of the hematoma, median (range)	810 (118, 2408)
Medical history, number (%)	
Active malignancy within 12 months	8 (24)
Dialysis	6 (18)
Surgery within the last 3 months	5 (15)
Cardiovascular event within the last 3 months	5 (15)
Steroid therapy	18 (54)
Oral antiplatelet use	5 (15)
Oral anticoagulant use	6 (18)
Heparin therapy	18 (52)
Laboratory value, median (range)	
Hemoglobin	7.5 (4.7, 15.4)
Platelets	15.8 (1.5, 59.4)
APTTR	1.7 (1.0, 5.9)
PT-INR	1.2 (1.0, 4.7)
Fibrinogen	272 (35, 502)
eGFR	36.9 (4.6, 145)

For all patients, the pre-treatment hemoglobin levels, platelet counts, and APTT ratios were obtained on the same day as the first TAE. Fibrinogen values were missing in one patient owing to a lack of preoperative testing. Fibrinogen values in two cases (10 and 11 days before, respectively), PT-INR (six days before), and eGFR (one day before) were obtained from tests conducted the day before the initial TAE.

Eight patients with COVID-19 complications were included: five on extracorporeal membrane oxygenation (ECMO) for severe respiratory failure and one who developed SMH shortly after weaning from ECMO. All eight patients were men and received steroid and anticoagulant therapy for COVID-19 pneumonia.

Comparison between non-rebleeding and rebleeding cases

**Figure 2:**
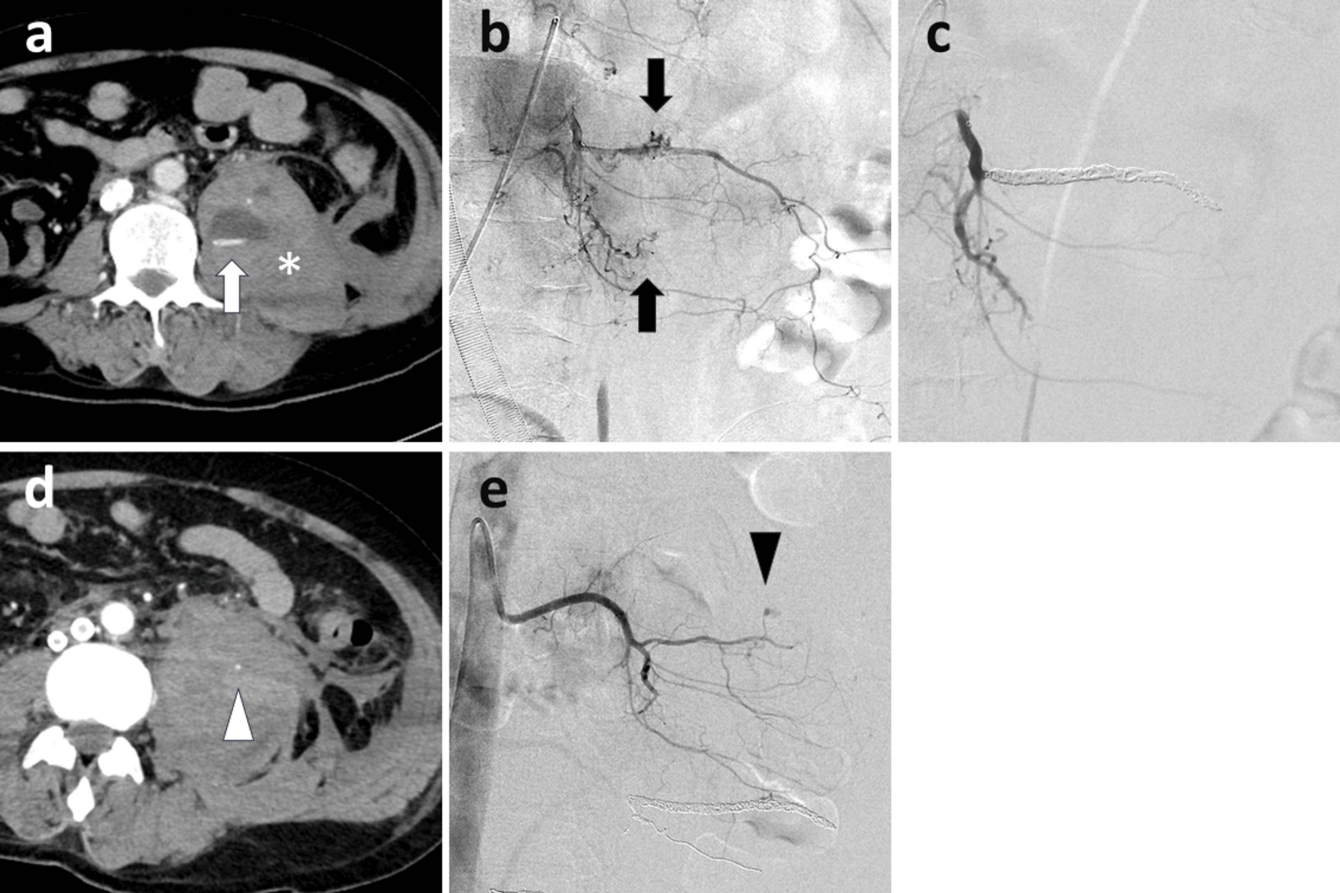
Imaging of a 50-year-old man who underwent extracorporeal membrane oxygenation owing to a severe coronavirus disease infection a: CECT reveals a hematoma (*) and contrast extravasation (white arrow) in the left psoas muscle. b: Left second lumbar arteriography demonstrates multiple sites of contrast medium extravasation (arrow). c: The second lumber artery is subsequently embolized using a coil and gelatine sponge. d: Two days after the initial transcatheter arterial embolization, CECT shows rebleeding (white arrowhead) in a region of the left psoas muscle that is a distinct area from the prior bleeding. e: Angiography of the first left lumbar artery shows new contrast medium extravasation at multiple sites (arrowhead), which was subsequently embolized using a gelatine sponge. CECT: contrast-enhanced computed tomography

Rebleeding occurred in nine patients (27.2%); among them, six, two, and one patients had one, two, and three rebleeds requiring repeat embolization, respectively. The time between the first and second bleeding events was one day in four patients, two days in two patients, and four, 12, and 22 days in one patient each. Only one patient experienced bleeding owing to the recanalization of an embolized vessel; all others experienced bleeding from non-embolized sites or through collateral channels. Figure 2a-2e shows an image of a patient treated for rebleeding.

**Figure 3:**
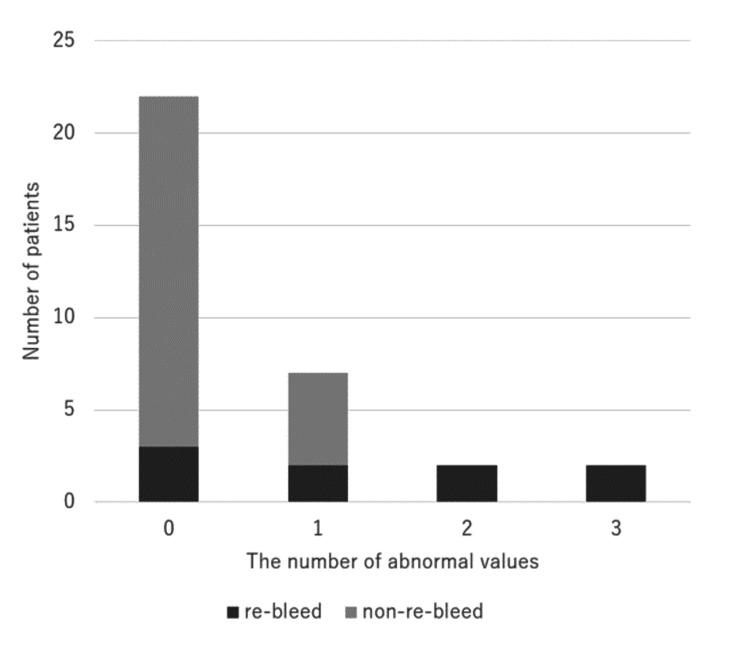
Distribution of rebleeding and non-rebleeding cases based on abnormal laboratory test results

Table 2 shows a comparison of the frequencies of potential risk factors between the non-rebleeding and rebleeding groups. The rebleeding group showed a higher proportion of patients with platelets < 5×10^4^/µL, fibrinogen < 150 mg/dL, and APTT ratio > 2.5 than the non-rebleeding group, with a statistically significant difference. Figure 3 shows the distribution of cases with rebleeding and non-rebleeding, classified based on the number of tests showing abnormal values among the three laboratory tests.

**Table 2:** Comparison of potential risk factors between the cases of non-rebleeding and rebleeding *In the non-rebleeding group, fibrinogen values were unavailable in one case. Std diff: standardized difference, APTTR: activated partial thromboplastin time ratio, PT-INR: prothrombin time/international normalized ratio, COVID-19: coronavirus disease 2019

Variables, number (%)	Non-rebleeding (n=24)	Rebleeding (n=9)	P-value	Std diff
Age > 75 years	11 (46)	1 (11)	0.11	0.83
Male sex	15 (63)	7 (78)	0.68	0.34
Gelatine alone	6 (25)	4 (44)	0.4	0.41
3-month history of cardiovascular event	4 (17)	1 (11)	1.0	0.16
3-month surgical history	5 (21)	0 (0)	0.29	0.72
12-month history of active malignancy	4 (17)	4 (44)	0.17	0.63
History of steroid therapy	11 (46)	7 (78)	0.13	0.70
Dialysis	3 (13)	3 (33)	0.31	0.51
COVID-19 infection	6 (25)	2 (22)	1.0	<0.1
Oral anticoagulant or heparin use	20 (83)	4 (44)	0.07	0.86
Hemoglobin level < 6 mg/dL	7 (29)	3 (33)	1.0	<0.1
Platelet count < 5×10^4^/μL	1 (4)	4 (44)	<0.05	1.06
Fibrinogen level < 150 mg/dL	2 (9)*	4 (44)	<0.05	0.89
PT-INR > 1.5	5 (21)	3 (33)	0.65	0.28
APTTR > 2.5	2 (8)	4 (44)	<0.05	0.89
Hematoma volume > 1000 mL	8 (33)	4 (44)	0.62	0.19

Anticoagulation and rebleeding

**Figure 4:**
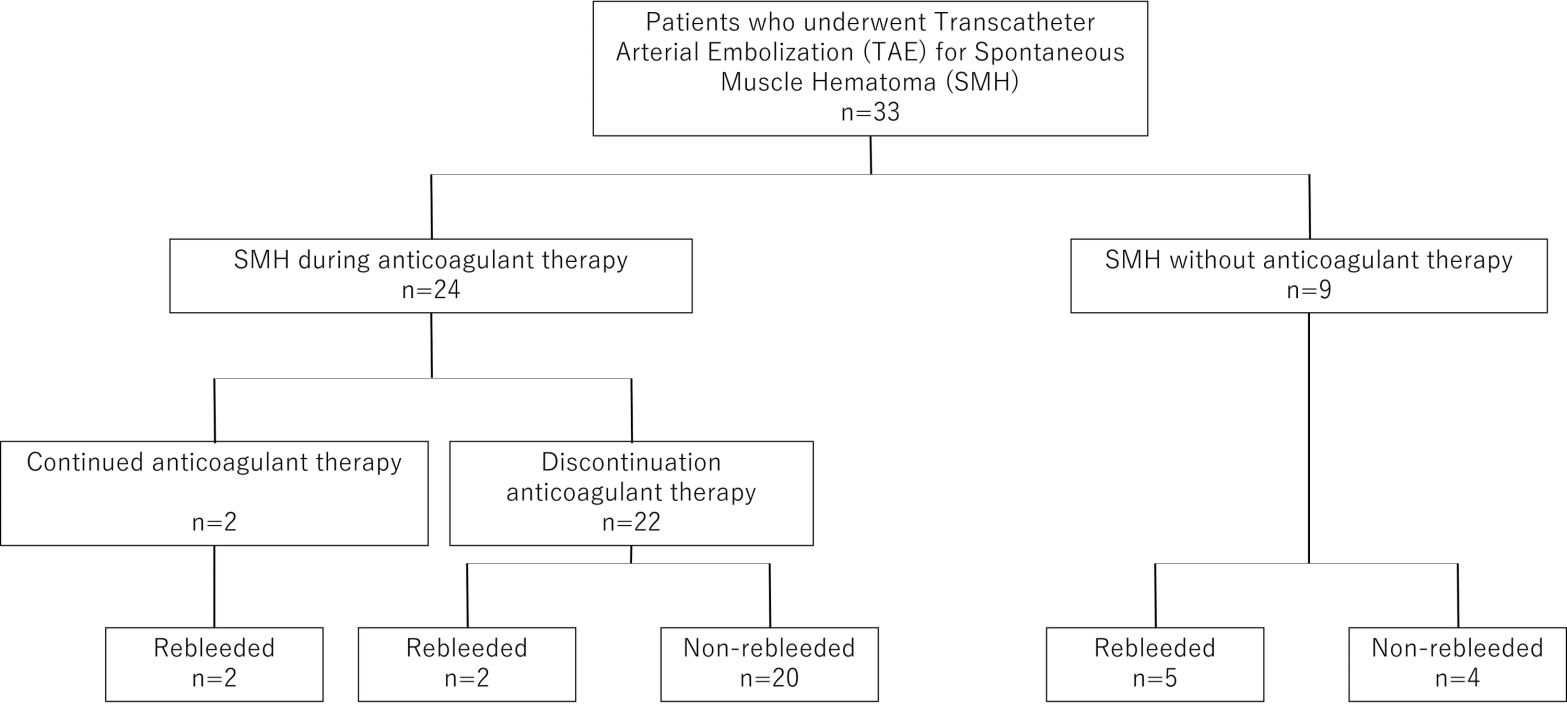
Flowchart of anticoagulants and rebleeding in patients who underwent transcatheter arterial embolization for spontaneous muscle hematoma TAE: transcatheter arterial embolization, SMH: spontaneous muscle hematoma

The proportion of patients receiving anticoagulation therapy in the rebleeding group was lower than that in the non-rebleeding group (Figure 4).

Rebleeding occurred in four of the 24 patients who developed SMH while on oral anticoagulants or heparin. However, two of them had concurrent antiphospholipid syndromes after mechanical valve replacement and acute strokes; thus, anticoagulant therapy was continued after the initial SMH based on clinical judgment, and both experienced rebleeding. Of the 22 patients who discontinued anticoagulation therapy after the initial SMH, rebleeding was observed in two (9%). In contrast, among the nine patients who developed SMH unrelated to anticoagulation therapy, rebleeding was observed in five (55.5%) (P=0.012).

Among nine patients with SMH unrelated to anticoagulation therapy, four had low fibrinogen levels, compared to two of 24 patients with SMH related to anticoagulation therapy. Low fibrinogen levels were significantly more common in the non-anticoagulation group (P=0.039). Four of the nine patients had a history of malignant disease within 12 months. Furthermore, most other patients had comorbidities not covered in this study protocol, such as a history of prostate cancer treatment, hemophilia detected after SMH, and acute respiratory distress syndrome caused by pneumonia.

Comparison between early mortality and survival

Table 3 compares the proportion of patients with factors potentially associated with bleeding between the early mortality and survival groups. The number of deaths due to all causes within one month was eight (24.2%). Among the factors evaluated, a significantly higher rate of early mortality within one month was observed in patients with active malignant complications than in those without (5/8, 63%). In contrast, patients who were retreated following rebleeding and those who did not rebleed had equivalent early mortality rates.

**Table 3:** Comparison of potential risk factors between the cases of early mortality and survival *In the non-rebleeding group, fibrinogen values were unavailable in one case. Std diff: standardized difference, APTTR: activated partial thromboplastin time ratio, PT-INR: prothrombin time/international normalized ratio, COVID-19: coronavirus disease 2019

Variables, number (%)	Survival (n=25)	Early mortality (n=8)	P-value	Std diff
Age > 75 years	7 (28)	5 (63)	0.11	0.74
Male sex	16 (64)	6 (75)	0.69	0.24
Gelatine alone	8 (32)	2 (25)	1.0	0.15
3-month history of cardiovascular event	5 (20)	0 (0)	0.30	0.71
3-month surgical history	4 (16)	1 (13)	1.0	0.1
12-month history of active malignancy	3 (12)	5 (63)	<0.05	1.23
History of steroid therapy	13 (52)	5 (63)	0.70	0.21
Dialysis	4 (16)	2 (25)	0.62	0.22
COVID-19 infection	5 (20)	3 (38)	0.37	0.39
Oral anticoagulant or heparin use	19 (76)	5 (63)	0.65	0.30
Hemoglobin level < 6 mg/dL	8 (32)	2 (25)	1.0	0.16
Platelet count < 5×10^4^/μL	3 (12)	2 (25)	0.57	0.34
Fibrinogen level < 150 mg/dL	4 (17)*	2 (25)	0.63	0.21
PT-INR > 1.5	5 (20)	3 (38)	0.37	0.39
APTTR > 2.5	4 (16)	2 (25)	0.62	0.22
Hematoma volume > 1000 mL	10 (40)	2 (25)	0.68	0.32
Rebleeding	7 (28)	2 (25)	1.0	<0.1

In addition, early mortality was higher among patients with COVID-19 infection than in those without, although the difference was not statistically significant. When the observation period was extended to three months, seven of eight (88%) patients with COVID-19 infection died, which was significantly higher than the nine of 25 (36%) non-COVID-19 patients who died (P<0.05).

**Figure 5:**
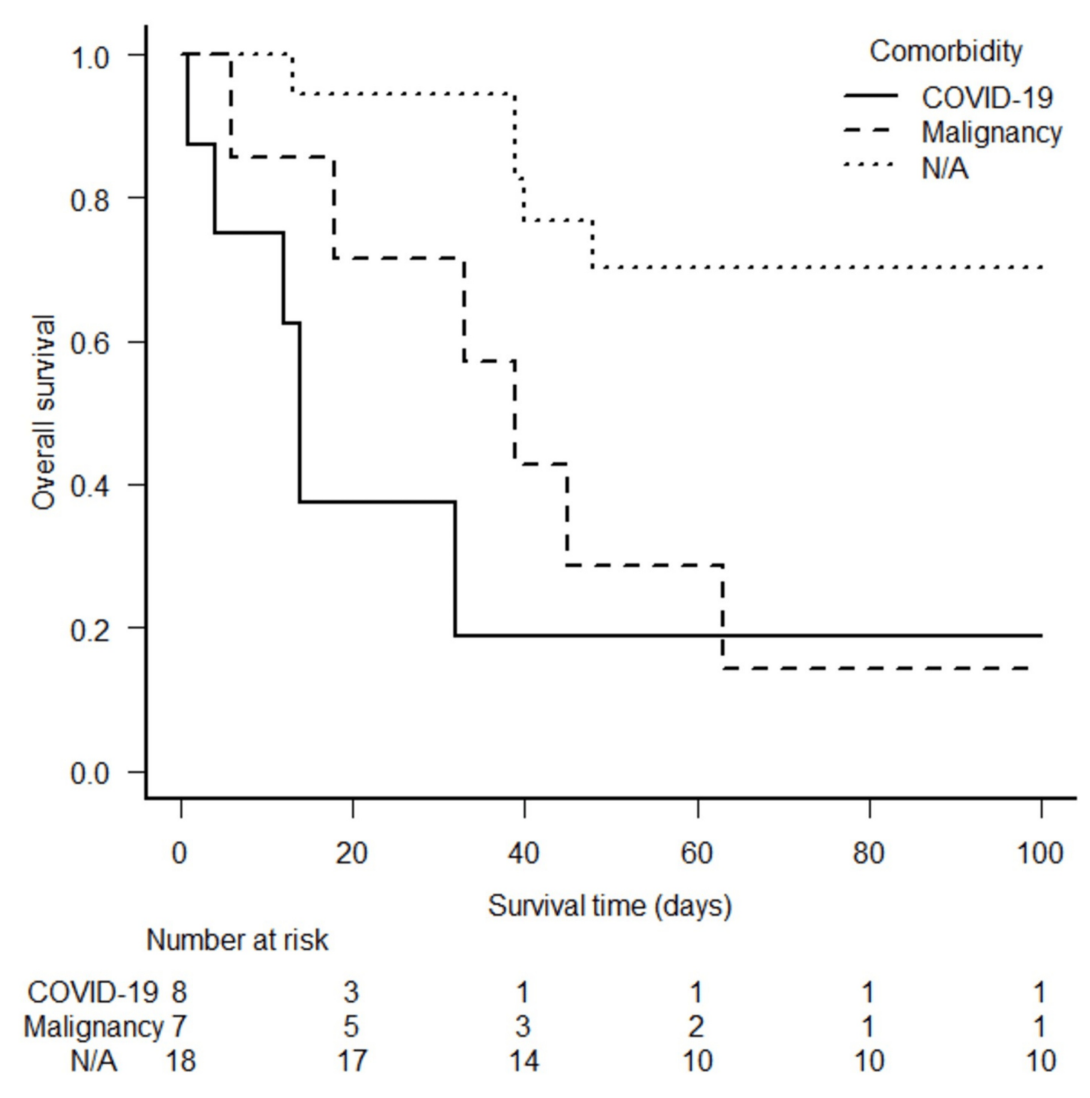
Kaplan-Meier curves of the overall survival rate of patients with a history of malignancy, with COVID-19, and without both comorbidities (N/A) COVID-19: coronavirus disease 2019, N/A: not applicable

Additionally, OS was compared according to the presence of the following comorbidities: a history of malignancy within 12 months and COVID-19 infection. One of the eight patients who developed SMH during treatment for COVID-19 infection had a history of lymphoma remission within one year and was assigned to the COVID-19 group. Figure 5 shows the Kaplan-Meier curves for the three groups of patients. The median follow-up period after the initial treatment was 48 (range: 1-3,232) days. Seventeen patients died during the follow-up period. Among them, 16 remained hospitalized after the initial embolization treatment and subsequently died (median: 25 days, range: 1-63 days). Of these, four had secondary hemorrhagic events in other organs, including intracranial hemorrhage, which was suggested to be related to death, while the remaining patients died of multiple organ failure without obvious hemorrhagic events. Another patient was discharged but died of sepsis 398 days later. The log-rank test with Bonferroni correction indicated significantly worse OS in patients with a history of malignancy (P=0.016) and in patients with COVID-19 (P=0.004) than in those without comorbidities.

## Discussion

In this retrospective study, the rebleeding rate within 30 days after TAE was 27.2% and the early mortality rate was 24.2%. Previous studies reported rebleeding rates of 14.3%-26.4% and early mortality rates of 26.8%-30.5%, which were generally consistent with our study's findings [1,5,14].

Although anticoagulant therapy is known to be a risk factor for the development of SMH, there have been no detailed reports on the risk factors involved in rebleeding after TAE for SMH. In this study, patients who developed rebleeding after TAE were more likely to have abnormal platelet, fibrinogen, and APTT values at initial onset than those who did not develop rebleeding after TAE. Additionally, most patients with rebleeding bled from a different site than the site of the first embolization. Dohan et al. reported that only one in six patients rebleed from the same vessel [5]. Abnormal hemostasis/coagulability may have caused rebleeding from an underlying vascular injury that was spontaneously hemostatic during the initial treatment or may have caused new bleeding. Therefore, careful follow-up is required if abnormal platelet, fibrinogen, or APTT values are observed at disease onset.

As in previous reports, many patients who developed SMH received anticoagulation therapy; however, a higher rebleeding rate was observed in patients who developed SMH unrelated to anticoagulation therapy and had various patient-related risk factors, including malignancy. Unlike anticoagulant-induced bleeding, these risk factors can cause long-term systemic effects, which are difficult to correct in the short term and can lead to rebleeding. Although there appears to be an overlap and confounding factor between multiple laboratory abnormalities and patient-related risk factors, along with patient-related risk factors being expected to vary depending on regional and institutional characteristics, the development of SMH in patients not taking anticoagulants may serve as a comprehensive risk factor for rebleeding.

The selection of embolic materials is another important consideration. Gelatin sponge particles, which were frequently used in this study, may not provide sufficient embolization in patients with coagulopathy. In such cases, permanent embolic materials, such as coils or NBCA, could be considered to achieve more effective hemostasis and reduce the risk of rebleeding. In this study, the rebleeding rate in patients with severe COVID-19 (22%) was comparable to that in other patients (25%). Steinberg et al. reported rebleeding rates of 32.3% and 4.5% in ECMO and non-ECMO patients, respectively, which is in line with our findings [17].

Barral et al. found that simplified acute physiology score II, hematoma volume, and retroperitoneal bleeding were predictors of early mortality in 112 patients with SMH [1]. In contrast, Decker et al. reported that only active bleeding, which is observable on CECT, was associated with early mortality, not hematoma volume [18]. In our study, all patients had active bleeding on angiography; additionally, the hematoma volume was not associated with early mortality. In contrast, the early mortality group had a significantly higher incidence of malignancy than the other group. Although malignancy complications have not been discussed in detail in previous reports, the reason for this difference may be the large number of cases with malignant tumor complications in our study, which may have biased the population.

The number of long-term cancer survivors has increased owing to advances in cancer treatment. However, they have increased various risks; accordingly, malignancy-related coagulopathy is an important prognostic factor. In a prospective study of 842 patients with VTE, Prandoni et al. reported that the cumulative major bleeding rates over 12 months were 12.4% in patients with cancer and 4.9% in those without cancer (hazard ratio: 2.2) [19]. Moreover, Sakamoto et al. reported a higher cumulative five-year incidence of major bleeding (26.6% versus 9.3%) and all-cause death (73.1% versus 14.6%) in patients with active cancer than in those without cancer in their multicenter VTE study [20].

There was no observed increase in early mortality in the group that received re-embolization for rebleeding, suggesting that embolization was effective in controlling SMH. There was no clear difference in the rebleeding rate or early mortality between patients with SMH complicated by severe COVID-19 and other patients. However, when the observation period was extended, the OS rate decreased in accordance with the severity of COVID-19 infection. It is noted that new variants of COVID-19 with different pathologies are emerging in rapid succession, and there is a possibility of selection bias as the cases discussed in this study were in the early stages of the pandemic. It should also be considered that the high mortality rate is not unrelated to bleeding status, as the majority of COVID-19 patients were treated with ECMO in this study. Additionally, given that only eight COVID-19 cases were included, it is possible that the role of COVID-19 in the outcomes of bleeding and mortality was not fully explored.

This study has several limitations. First, this was a long-term retrospective study conducted at a single center, without considering potential changes in social and technological factors. Second, the limited sample size, especially the small number of patients experiencing rebleeding, reduced the statistical power and prevented the establishment of causal relationships and generalizations. Third, the inclusion criteria were limited to patients undergoing TAE, potentially excluding those managed conservatively and skewing the findings toward more severe presentations of SMH. Additionally, unexamined risk factors may have influenced the outcomes. Our conclusions are further constrained by the reliance on univariate analyses. Therefore, future research employing multivariate analysis to compare OS rates in prospective studies is recommended.

## Conclusions

This study indicated that hemostatic and coagulation factors, such as the platelet count, fibrinogen level, and APTT, may influence the outcome of rebleeding. Additionally, SMH unrelated to anticoagulation therapy exhibited a higher rebleeding rate compared to SMH with anticoagulation therapy. Early rebleeding treated with TAE does not affect early mortality rates. However, severe comorbidities such as malignancy or severe COVID-19 infection may impact mortality. Therefore, proactive identification of high-risk patients could potentially lead to the development of enhanced treatment strategies and clinical outcomes.
